# Diagnose und Therapie glomerulärer Erkrankungen mit einem membranoproliferativen Läsionsmuster (MPGN) – 2023

**DOI:** 10.1007/s00508-023-02264-7

**Published:** 2023-09-20

**Authors:** Michael Rudnicki, Martin Windpessl, Kathrin Eller, Balazs Odler, Philipp Gauckler, Irmgard Neumann, Emanuel Zitt, Heinz Regele, Andreas Kronbichler, Karl Lhotta, Marcus D. Säemann

**Affiliations:** 1 Department Innere Medizin 4 (Nephrologie und Hypertensiologie), Medizinische Universität Innsbruck, Anichstraße 35, 6020 Innsbruck, Österreich; 2 Abteilung für Innere Medizin IV, Klinikum Wels-Grieskirchen, Wels, Österreich; 3 Medizinische Fakultät, JKU, Linz, Österreich; 4 Klinische Abteilung für Nephrologie, Abteilung für Innere Medizin III (Nephrologie, Dialyse und Hypertensiologie), Medizinische Universität Graz, Graz, Österreich; 5 Department Innere Medizin IV (Nephrologie und Hypertensiologie), Medizinische Universität Innsbruck, Innsbruck, Österreich; 6 Vasculitis.at, Wien, Österreich; 7 Immunologiezentrum Zürich (IZZ), Zürich, Schweiz; 8 Abteilung für Innere Medizin III (Nephrologie, Dialyse und Hypertensiologie), Akademisches Lehrkrankenhaus Feldkirch, Feldkirch, Österreich; 9 Klinisches Institut für Pathologie, Medizinische Universität Wien, Wien, Österreich; 10 6. Medizinische Abteilung mit Nephrologie & Dialyse, Klinik Ottakring, Wien, Österreich; 11 Medizinische Fakultät, SFU, Wien, Österreich

**Keywords:** MPGN Immune-complex Glomerulonephrits, C3 Glomerulonephritis, Dense Deposit Diesease, Supportiv-Therapie, Rituximab, Eculizumab, Immune-complex glomerulonephrits, C3 glomerulonephritis, Dense deposit disease, Supportive treatment, Rituximab, Eculizumab

## Abstract

Die membranoproliferative Glomerulonephritis (MPGN) repräsentiert eine heterogene Gruppe von Erkrankungen. Das gemeinsame Merkmal eines membranoproliferativen Läsionsmusters in der Histologie der Nierenbiopsie kann sowohl idiopathisch/primär auftreten, als auch – wesentlich häufiger – eine sekundäre Ursache haben. Die historische licht- und elektronenmikroskopische Einteilung in MPGN Typ I bis III wurde weitgehend verlassen und in den letzten Jahren durch eine Pathogenese-orientierte Einteilung ersetzt. Von einer Immunkomplex-GN (IK-GN) spricht man beim Vorliegen einer MPGN mit C1q, C3 und/oder C4 Ablagerungen, während eine MPGN mit dominanten C3-Ablagerungen als C3-Glomerulopathie (C3G) bezeichnet wird. Diese wird wiederum in eine C3-Glomerulonephritis (C3GN) und eine dense-deposit disease (DDD) eingeteilt. Diese Diagnosen können nur durch eine Nierenbiopsie gestellt werden. Mögliche Ursachen für eine MPGN sind chronische Infektionen (v. a. Hepatitis B und C, bakterielle Infektionen, Infektionen mit Protozoen). Autoimmunerkrankungen (v. a. Lupus, rheumatoide Arthritis) oder Malignome (v. a. hämatologische maligne Erkrankungen). Insbesondere im Falle einer C3G wird auch eine umfassende Abklärung des Komplementystems empfohlen. Therapeutische Entscheidungen sind aufgrund der niedrigen Inzidenz und des heterogenen klinischen Erscheinungsbildes einer MPGN individuell zu treffen, eine optimale generelle Therapie ist unbekannt. Im Falle einer identifizierten Ursache einer MPGN wird prinzipiell empfohlen diese zu behandeln, ebenso sollte die supportive Therapie, wie auch bei anderen Glomerulonephritiden optimiert werden. Bei anhaltender signifikanter Proteinurie und einer eGFR > 30 ml/min/1,73 m^2^ wird eine Therapie mit systemischen Steroiden und Mycophenolat Mofetil empfohlen. Weitere Therapieoptionen sind Rituximab und Eculizumab. Eine rapid-progressive MPGN sollte wie eine ANCA-assoziierte Vaskulitis therapiert werden. Die Rekurrenzraten nach einer Nierentransplantation sind sehr hoch und therapeutisch herausfordernd.

## Abstract (en)

Membranoproliferative glomerulonephritis (MPGN) represents a heterogeneous group of diseases. The common feature of a membranoproliferative lesion pattern in the kidney biopsy can either be idiopathic/primary or—much more frequently—have a secondary cause. The historical classification into MPGN types I to III has largely been abandoned and replaced in recent years by a pathogenesis-oriented classification. A MPGN with C1q, C3 and/or C4 deposits on light microscopy is referred to as immune complex GN (IC-GN), while a MPGN with dominant C3 deposits is referred to as C3 glomerulopathy (C3G). C3G is further divided into C3 glomerulonephritis (C3GN) and dense deposit disease (DDD). These diagnoses can only be made by a kidney biopsy. Possible causes of MPGN are chronic infections (especially hepatitis B and C, bacterial infections, infections with protozoa), autoimmune diseases (especially lupus, rheumatoid arthritis) or malignancies (especially hematological malignancies). Particularly in the case of C3G a comprehensive analysis of the complement system components is strongly recommended. Due to the low incidence and the heterogeneous clinical appearance of MPGN therapeutic decisions must be made individually; an optimal general therapy is unknown, except that supportive treatment as with other glomerular diseases should be optimized. In the case of a secondary MPGN it is generally recommended to treat the potential cause of the MPGN. If significant proteinuria persists and eGFR remains > 30 ml/min/1.73 m^2^, treatment with systemic steroids and mycophenolate mofetil is recommended. Other treatment options on an individual level after evaluation and discussion of the risk-benefit ratio with the patient are rituximab and eculizumab. Rapidly progressive MPGN should be treated like ANCA-associated vasculitis. The recurrence rates after kidney transplantation are very high and treatment is challenging.

## Hintergrund

Die membranoproliferative Glomerulonephritis (MPGN) repräsentiert eine heterogene Gruppe von Erkrankungen, deren gemeinsames Merkmal ein membranoproliferatives Läsionsmuster in der Histologie der Nierenbiopsie darstellt. Ein zentrales Merkmal der Läsion, der Umbau der glomerulären Kapillarwände, lässt auf eine Störung der glomerulären Endothelzellen schließen, deren Ursache allerdings sehr heterogen ist.

Aufgrund signifikanter Fortschritte im Verständnis der Pathogenese dieser Nierenpathologie, insbesondere aufgrund der Erkenntnis, dass pathologische Aktivierungen des Komplementsystems maßgeblich zur MPGN beitragen können, haben sich die Einteilung, die differentialdiagnostische Abklärung der Ursachen und die therapeutischen Ansätze zur Behandlung der MPGN im letzten Jahrzehnt deutlich gewandelt [1, 2]. Die Identifikation einer Ursache für die glomeruläre Ablagerung von Immunglobulinen und/oder Komplementfaktoren stellt den ersten und maßgeblichen Schritt in der Abklärung der MPGN dar. Die Therapie hängt in der Regel von der zugrundeliegenden Pathogenese ab (siehe unten). Neben einer ursachenspezifischen Therapie ist in manchen Fällen eine immunsuppressive Therapie indiziert [3–6], jedoch ist aufgrund der niedrigen Inzidenz, kleiner Patient:innenzahlen in wenigen prospektiven Studien und der Heterogenität dieser Patient:innenkohorten eine solide Therapieempfehlung nur schwer möglich. Aus diesen Gründen ist eine immunsuppressive Therapie immer individualisiert durchzuführen. Wenn möglich sollen Patient:innen mit glomerulären Erkrankungen mit einem membranoproliferativen Läsionsmuster in Studien eingeschlossen und gemeinsam mit Zentren mit entsprechender Erfahrung in der Abklärung und Therapie dieser Erkrankungen betreut werden.

## Klinische Symptomatik und Diagnostik

Üblicherweise findet sich bei einer aktiven MPGN ein „nephritisches Bild“, d. h. Hämaturie mit oder ohne Akanthozyten und eine variabel ausgeprägte Proteinurie, durchaus bis hin zum nephrotischen Syndrom reichend. In manchen Fällen, insbesondere wenn die Ursache einer MPGN schon länger zurückliegt, zeigt sich kein aktives Sediment, d. h. keine Hämaturie, sondern lediglich eine Proteinurie. Die Nierenfunktion kann normal oder eingeschränkt sein, eine Hypertonie kann ebenso vorliegen. Eine Komplementaktivierung (d. h. erniedrigtes C3 und/oder C4 im Blut) kann bei allen Formen einer MPGN vorliegen, allerdings kann man bei normalen Serum C3- und/oder C4-Konzentrationen eine Komplement-mediierte MPGN nicht ausschließen [7, 8]. Insgesamt ist die Präsentation unspezifisch und von anderen Glomerulonephritiden nicht zu unterscheiden, weswegen die Diagnose einer MPGN schlussendlich nur mit einer Nierenbiopsie möglich ist [9].

## Historische Nomenklatur

Typischerweise findet sich bei der MPGN im lichtmikroskopischen Bild eine Expansion der mesangialen Matrix, eine Hyperzellularität und eine akzentuierte Lobulierung des glomerulären Schlingenkonvolutes sowie ein Umbau der glomerulären Kapillarwände (*„double contour“, „tram-track“*). Letzteres, sowie die Hyperzellularität sind die namengebenden morphologischen Charakteristika der Läsion, die allerdings nicht auf einer einheitlichen Pathogenese beruhen. Die inzwischen überholte Unterteilung in Typ I, II und III basiert nur auf morphologisch-deskriptiven Kriterien durch die Lokalisation elektronendichter Ablagerungen im elektronenmikroskopischen Bild [10]:

MPGN Typ I: Die Ablagerungen finden sich hauptsächlich *subendothelial* und *mesangial*. In der Immunfluoreszenz bestehen diese aus Immunglobulinen und C3.

MPGN Typ II: Dieser Typ der MPGN wird auch „*dense deposit disease (DDD)*“ genannt: Es handelt sich hierbei um keine Immunkomplex-Erkrankung, sondern die DDD wird durch elektronendichte Ablagerungen *innerhalb der Basalmembran charakterisiert*, welche in der Immunfluoreszenz als Bestandteile des Komplementsystems (vorwiegend Fragmente von C3) identifiziert werden.

MPGN Typ III: Bei diesem Typ der MPGN finden sich Ablagerungen sowohl *subepithelial *als auch *subendothelial*, also auf beiden Seiten der Basalmembran.

Diese historische Einteilung der MPGN wurde mittlerweile durch eine pathogenetisch orientierte Klassifikation ersetzt.

## Pathogenese-orientierte Einteilung der MPGN

**Figure 1:**
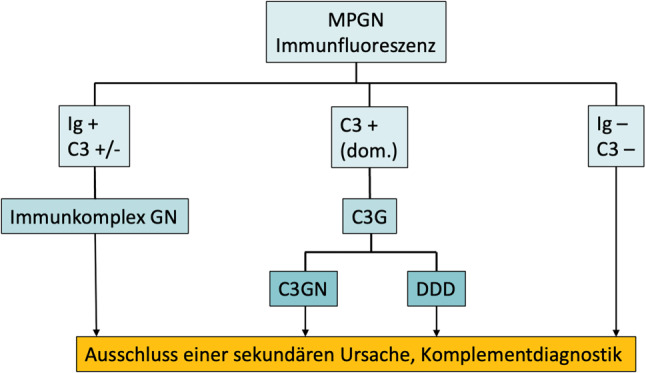

Erkenntnisse über die pathologische Aktivierung des Komplementsystems bei einem Teil der MPGN-Fälle hat zu einer neuen Pathogenese-orientierten Einteilung dieser Erkrankungen geführt [9, 11]. Werden membranoproliferative Läsionsmuster in der Lichtmikroskopie identifiziert, dann folgt die weitere (grobe) Einteilung den Ergebnissen der Immunfluoreszenz (Abb. 1):

- Eine Immunglobulin-dominante MPGN mit (weniger dominanten) C1q, C3 und/oder C4 Ablagerungen wird als **Immunkomplex-GN (IK-GN)** bezeichnet. Die Immunkomplexe können monoklonal oder polyklonal sein.
- Eine MPGN mit dominanten C3-Ablagerungen wird als **C3-Glomerulopathie** (C3G) bezeichnet.
- MPGN ohne Immunglobulin- und Komplement Ablagerungen.

## Differentialdiagnostische Abklärung

### Immunkomplex-GN (IK-GN)

Bei einer IK-GN dominiert ein membranoproliferatives Läsionsmuster mit der glomerulären Ablagerung von Immunkomplexen, die durch den immunhistologischen Nachweis von Immunglobulinen und Komplementkomponenten definiert sind. Ursache ist häufig eine chronische Antigenämie mit oder ohne zirkulierenden Immunkomplexen. Folgende mögliche Ursachen sollten erkannt bzw. ausgeschlossen werden ([2]; Tab. 1; weder die Tab. 1 noch der Text erheben einen Anspruch auf Vollständigkeit, die häufigsten bekannten Ursachen sind angegeben):

- Infektionen: Hepatitis B, Hepatitis C (inkl. Kryoglobulinämie), Endokarditis, Abszesse, chronische Streptokokken- und Staphylokokken-Infektionen (ASLO, ASTA), Protozoeninfektionen (Malaria, Schistosomiasis, Mycoplasmosen, Leishmaniose, etc.)
- Autoimmunerkrankungen: Systemischer Lupus erythematodes (SLE, v. a. Klasse IV Lupus Nephritis), mixed connective tissue disease (MCTD), Sjögren Syndrom, Rheumatoide Arthritis (hier vor allem lange Erkrankungsdauer, mit langjähriger chronischer Erkrankungsaktivität oder medikamentös-toxisch)
- Malignome: Altersentsprechendes Malignom-Screening, insbesondere auf hämatologische Erkrankungen (siehe unten)

**Table 1:** 

Infektionen	Autoimmunerkrankungen	Malignome	Idiopathisch
Hepatitis B Hepatitis C (± Kryoglobulinämie) Endokarditis Abszesse Staphylokokken Streptokokken Malaria Schistosomiasis Mycoplasmen Leishmaniose	SLE MCTD RA Sjögren’s Syndrom	v. a. hämatologische Malignome	Keine Ursache fassbar

*SLE* Systemischer Lupus erythematodes, *MCTD* mixed connective tisue disease, *RA* Rheumatoide Arthritis

Einen Sonderfall einer MPGN stellt eine proliferative Glomerulonephritis mit monoklonalen Immunglobulinablagerungen (PGNMID) dar, welche sich gelegentlich bei Patient:innen mit monoklonalen Gammopathien findet. Tritt eine proliferative B‑Zell- oder Plasmazellerkrankung, die *per se* keine therapeutische Konsequenz hätte, in Assoziation mit einer MPGN auf, so spricht man von einer monoklonalen Gammopathie mit renaler Signifikanz (MGRS), welche dann sehr wohl eine therapeutische Konsequenz nach sich zieht [12]. Insbesondere bei PatientInnen ≥50 Jahre mit der erstmaligen Diagnose einer MPGN bzw. IK-GN sollte deshalb eine extensive, v. a. hämatologische Abklärung, durchgeführt werden, vorzugsweise in interdisziplinärer Zusammenarbeit mit der Hämatologie.

Ist die oben genannte Abklärung betreffend die Ätiologie einer IK-GN negativ, so wird eine erweiterte Evaluierung möglicher Komplementdysregulationen empfohlen, analog zur Abklärung bei einer C3 Glomerulopathie (siehe unten, Tab. 2).

**Table 2:** 

*Komplementfaktoren*	C3, C4, C1q, Faktor I, Faktor H, Faktor B, Properdin, lösliches C5b‑9 (MAC, TCC)
*Autoantikörper*	C3 Nef, C4 Nef, C5 Nef, anti Faktor H, anti Faktor B
*Genetische Analyse*	C3, CFH, CFI, CFB, CFHR1‑5, MCP

*MAC* membrane attack complex = TCC terminaler Komplementkomplex, *Nef* Nephritis Faktor, *CFH, CFI, CFB* Komplementfaktor H, I, B, *CFHR1‑5* complement factor H‑related protein, *MCP* membrane cofactor protein

### C3 Glomerulopathie (C3G)

Sind in der Immunhistochemie/Immunfluoreszenz die glomerulären C3-Ablagerungen mindestens zwei Größenordnungen dominanter als die Immunglobulinablagerungen, so spricht man von einer C3-Glomerulopathie (C3G) [13]. Anhand der Immunhistochemie/Immunfluoreszenz und der Elektronenmikroskopie wird die C3G in die C3-Glomerulonephritis (C3GN) und die Dense-Deposit-Disease (DDD) eingeteilt [14]. Ähnlich wie bei der IK-GN sollten zuerst eine Infektions-assoziierte GN und eine MGRS ausgeschlossen werden. Insbesondere (jedoch nicht nur) bei Patient:innen > 50 Jahre, bei denen eine C3G diagnostiziert wurde, liegt eine monoklonale Gammopathie 10–20 × häufiger vor als in einer altersgleichen Allgemeinbevölkerung. Es wird angenommen, dass eine C3G aus einer Dysregulation des Komplementsystems resultiert, hauptsächlich durch eine pathologische Aktivierung des alternativen Komplementweges. Erniedrigte C3 und C4 Spiegel liegen jedoch in weniger als 50 % der Fälle vor, so dass bei einer C3G empfohlen wird – unabhängig von der Serumkonzentration der C3 und C4 Spiegel – eine umfassende Abklärung des Komplementsystems durchzuführen (Tab. 2; [1, 2]). Obwohl ein MPGN-Muster typisch für die C3G ist, manifestiert sich diese häufig auch mit anderen glomerulonephritischen Läsionsmustern.

### MPGN ohne IK- oder Komplementablagerungen

Ein der MPGN-ähnliches Läsionsmuster kann auch bei chronischer thrombotischer Mikroangiopathie (TMA; z. B. bei einem hämolytisch-urämischen Syndrom oder einer thrombotisch-thrombozytopenen Purpura), bei einem Antiphospholipidsyndrom, einer Nephropathie assoziiert mit einer Knochenmarkstransplantation, einer Strahlentherapie-assoziierten Nephritis, einer chronischen antikörpermediierten Transplantatabstoßung oder bei einer malignen Hypertonie auftreten (Tab. 3). Hierbei kommt es primär zu einer Schädigung des (glomerulären) Endothels mit nachfolgendem reparativen Umbau der glomerulären Kapillarwände, was in einem membranoproliferativen Läsionsmuster mündet. IK- oder Komplementablagerungen fehlen für gewöhnlich, ebenso finden sich keine elektronendichten Depots in der Elektronenmikroskopie [2] und auch die glomeruläre Zellvermehrung ist, außer bei der chronischen antikörpermediierten Transplantatabstoßung, nur gering ausgeprägt oder fehlt völlig.

**Table 3:** 

Abheilung einer thrombotischen Mikroangiopathie (HUS/TTP)
Antiphospholipid-Syndrom
Strahlennephritis
Nephropathie assoziiert mit Knochenmarkstransplantation
Antikörpermediierte Transplantatabstoßung
Maligne Hypertonie

*HUS* Hämolytisch-urämisches Syndrom, *TTP* Thrombotisch-thrombozytopenische Purpura

## Therapie

Aufgrund der niedrigen Inzidenz der MPGN, des heterogenen klinischen Erscheinungsbilds und der vielfältigen Unterschiede in der Pathogenese sind generelle Empfehlungen z. B. für eine breite oder aggressive Immunsuppression nicht mehr aktuell [15]. Therapieentscheidungen bei Patient:innen mit einer MPGN müssen immer höchst individuell getroffen werden. Es existieren keine Daten aus prospektiven und randomisiert-kontrollierten Studien, sondern hauptsächlich Daten aus kleinen retrospektiven oder bestenfalls prospektiven nicht kontrollierten Kohortenstudien. Zudem führt der Zuwachs an Erkenntnissen über die Pathogenese dieser Erkrankung(en) zu einer diversifizierten Sicht auf die Heterogenität dieser historischen Kohortenstudien. Therapieentscheidungen basieren auf der Ursache, dem Ausmaß der Proteinurie und der Nierenfunktion bzw. der Nierenfunktionsdynamik sowie dem klinischen Gesamtbild betroffener PatientInnen.

### Therapie der Ursache

Die optimale Behandlung von Patient:innen mit einer IK-GN ist nicht bekannt. Es wird empfohlen, die potenzielle Ursache einer IK-GN zu behandeln. Zu den häufigsten Ursachen einer IK-GN zählen Infektionen mit Hepatitis B- und C‑Viren, in diesen Fällen ist die Therapie entsprechend antiviral, eine immunsuppressive Therapie ist nicht nur nicht indiziert, sondern für die PatientInnen auch potenziell bedrohlich. Ausnahmen stellen die HCV-assoziierte Kryoglobulinämie und die rapid-progressive Glomerulonephritis (RPGN) dar, welche immunsuppressiv behandelt werden sollten. Patient:innen, bei denen eine bakterielle und oder parasitäre Ursache einer MPGN identifiziert worden ist (siehe Tab. 1), sollten entsprechend antimikrobiell therapiert werden.

Wurde eine Autoimmunerkrankung wie z. B. ein SLE als Ursache einer IK-GN diagnostiziert, so sollten die PatientInnen gemäß den Empfehlungen zur Therapie dieser Autoimmunerkrankung behandelt werden.

Ist eine MPGN mit einer hämatologischen malignen Erkrankung, wie z. B. mit einer Waldenström Makroglobulinämie, einem Multiplen Myelom oder einer chronisch lymphozytären Leukämie (CLL) assoziiert, so folgt die Behandlung den entsprechenden Empfehlungen zur Behandlung des Malignoms. Im Falle eines nicht-malignen oder prämalignen Plasmazell- oder B‑Zell-Klons spricht man von einer MGRS bzw. von einer PGNMID. In solchen Fällen sollte der entsprechende Plasmazell- oder B‑Zell-Klon therapiert werden, ähnlich wie bei einem Multiplen Myelom, einer Waldenström Makroglobulinämie oder einer CLL [2, 12].

### Supportive Therapie

Alle Patient:innen mit einer IK-GN sollten eine supportive Therapie entsprechend den allgemeinen Empfehlungen zur Behandlung von Glomerulonephritiden erhalten. Es gibt keine Evidenz, dass PatientInnen mit einer idiopathischen IK-GN mit einer stabilen eGFR und einer nicht-nephrotischen Proteinurie von einer Immunsuppression profitieren würden. Historische Daten zeigen, dass Kinder mit einer MPGN, einer nicht-nephrotischen Proteinurie, normalen Blutdruck und einer normalen Nierenfunktion eine exzellente renale Prognose haben [16]. Diese PatientInnen sollten regelmäßig kontrolliert werden, da es im Verlauf zu einer Dynamik sowohl der Proteinurie als auch der Nierenfunktion kommen kann. Es gibt jedoch keine absoluten Grenzwerte der Proteinurie, der Nierenfunktion oder der Hämaturie, die als Aktivitätsmarker angewandt werden können, um eine Therapieentscheidung bzgl. einer immunsuppressiven Therapie zu treffen. Es ist sinnvoll, eine Nierenbiopsie zu wiederholen um die Aktivität und Chronizität der MPGN zu reevaluieren. Im Falle einer aktiven Glomerulonephritis kann eine immunsuppressive Therapie individuell mit dem Patient:innen besprochen und eine gemeinsame Entscheidung getroffen werden (*„informed decision making“*).

### Immunsuppression

Der klinische Verlauf bei PatientInnen mit einer MPGN kann sehr heterogen sein. Phasen einer milden Erkrankung mit erhaltener Nierenfunktion und sub-nephrotischer Proteinurie mit oder ohne Hämaturie (als Zeichen der Aktivität der MPGN) können stabil oder langsam chronisch progredient verlaufen; manchmal können Phasen einer rapiden Verschlechterung der Nierenfunktion auftreten; ein nephrotisches Syndrom kann auch bei einer fortgeschrittenen tubulointerstitiellen Fibrose auftreten, auch ohne oder nur mit einer geringen glomerulären Inflammation. Umgekehrt kann eine ausgesprochene glomeruläre Inflammation vorliegen, während die Proteinurie nicht-nephrotisch ist (< 3 g/Tag), der Patient also möglicherweise von einer immunsuppressiven Therapie profitieren könnte. Es ist klinisch oft unklar, wann welche Aktivität der MPGN vorliegt, was die Heterogenität der Studienergebnisse der Therapiestudien erklärt. Da die optimale Therapie unklar ist, wird empfohlen jede immunsuppressive Therapie mit den PatientInnen zu diskutieren [2].

**Figure 2:**
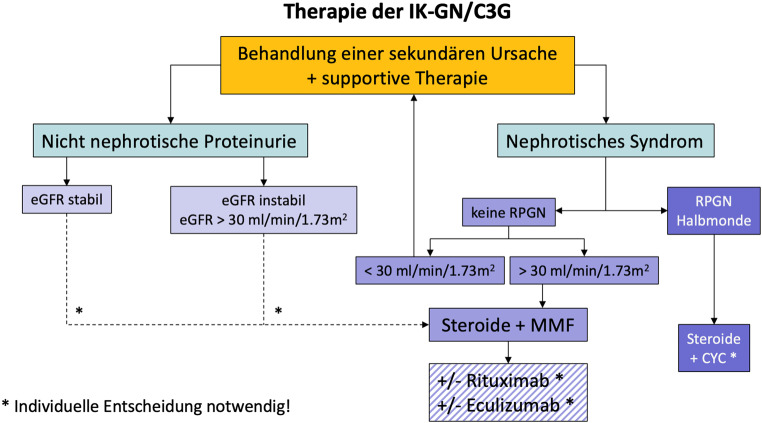

Üblicherweise ist die Indikation zum Beginn einer immunsuppressiven Therapie bei Patient:innen mit MPGN eine nephrotische Proteinurie (> 3 g/Tag). Möglicherweise profitieren jedoch v. a. Patient:innen mit einer C3G auch bei niedrigerer Proteinurie (> 1 g/Tag) und einer Hämaturie von einer immunsuppressiven Therapie [17]. Diese Empfehlung basiert auf retrospektiven Kohorten und der Extrapolation von Daten von anderen proliferativen Glomerulonephritiden [5, 6, 18]. Kontrollierte Daten zur MPGN gibt es nicht. Bei einer eGFR > 30 ml/min/1,73 m^2^ ohne Hinweis für eine RPGN wird in den Richtlinien eine Therapie mit Prednison (oder Äquivalent) mit 1 mg/kg pro Tag für 12–16 Wochen empfohlen. Die Evidenz stammt aus Studien mit Kindern mit MPGN, belastbare Daten zu Steroid bei erwachsenen Patient:innen mit MPGN gibt es nicht. Wir empfehlen jedoch als Erstlinientherapie bei idiopathischer IK-GN und C3G eine Therapie mit Mycophenolat Mofetil (MMF) 1–2 g pro Tag in Kombination mit Steroid (Abb. 2). Diese Empfehlungen basieren auf den Ergebnissen von 2 retrospektiven Studien zur C3G bei Erwachsenen, können jedoch auch für eine IK-GN angewandt werden [5, 6]. In der Arbeit von Rabasco et al. war eine Kombination von MMF mit Steroiden mit einer besseren renalen Prognose assoziiert als Steroide allein oder Steroide mit Cyclophosphamid. In der retrospektiven Arbeit von Avasare et al. war die Rate an klinischen Remissionen unter MMF in Kombination mit Steroiden höher, verglichen mit Steroiden allein, Cyclophosphamid mit Steroiden, Calcineurin-Inhibitoren oder Rituximab.

MMF sollte mit 500 mg zweimal täglich begonnen und auf 1000 mg zweimal täglich hinauftitriert werden. Wir empfehlen den Beginn mit einer reduzierten Dosis von 20 mg Prednison (oder Äquivalent, d. h. 16 mg Methylprednisolon oder 20 mg Prednisolon) und eine langsame Reduktion bis zu einer Erhaltungsdosis von 2,5–5 mg pro Tag. Die optimale Therapiedauer ist nicht bekannt. In der Literatur wird eine Therapiedauer von 12–18 Monaten empfohlen (Expertenmeinungen, [1]).

In 67–86 % der Fälle kommt es zu einer partiellen oder kompletten Remission, allerdings ist die Relapsrate nach Reduktion oder Absetzen von MMF relativ hoch (38–50 %).

Kommt es innerhalb von 6–12 Monaten zu keiner signifikanten Verbesserung von Nierenfunktion, Proteinurie oder Hämaturie, so sollte eine erneute Nierenbiopsie in Betracht gezogen werden. Zeigen sich in der Biopsie erneut aktive Läsionen, so empfehlen wir eine Therapieumstellung auf Rituximab. Daten zur Effektivität von Rituximab bei IK-GN und C3G beziehen sich auf Fallberichte und kleine retrospektive Fallserien [19]. Rituximab sollte in der Dosis von 1 g gegeben werden mit der Wiederholung nach 14 Tagen. Bei stabiler Nierenfunktion und Abnahme der Proteinurie sollte die erneute Gabe von Rituximab nach 6 Monaten überlegt werden [2].

Patient:innen mit einer eGFR < 30 ml/min/1,73 m^2^ sollten in den meisten Fällen supportiv therapiert werden, außer es besteht eine andere als die renale Indikation für eine immunsuppressive Therapie.

PatientInnen mit einem rapid-progressiven Verlauf einer MPGN (RPGN; üblicher- aber nicht notwendigerweise mit Halbmonden assoziiert) sollten mit Hochdosissteroid und Cyclophosphamid analog dem Schema für eine ANCA-assoziierte Vasculitis behandelt werden [2].

PatientInnen mit einer IK-GN aufgrund eines Kryoglobulinämie-Syndroms, welches häufig mit einer Hepatitis C Virusinfektion assoziiert ist, sollten nicht nur antiviral, sondern auch immunsuppressiv mit Hochdosis-Steroiden und Rituximab therapiert werden [20, 21]. In seltenen, lebensbedrohlichen Fällen ist die Durchführung eines Plasmaaustauschs (PLEX) indiziert [22].

### Komplementhemmung

Aufgrund der in vielen Fällen nachgewiesenen Assoziation zwischen pathologischen Dysregulationen (Aktivierungen) des Komplementsystems und v. a. der C3G, erscheint es naheliegend, dass eine Komplementhemmung mit dem anti-C5 Antikörper Eculizumab effektiv sein müsste. Die Daten aus wenigen Fallberichten und Fallserien sind jedoch widersprüchlich [3, 4]. Bei Nicht-Ansprechen auf immunsuppressive Therapien kann eine Therapie mit Eculizumab überlegt werden [2]. Serum C5b-9-Spiegel sind keine validen Biomarker für ein Therapieansprechen und sollten nicht für eine Entscheidung für oder gegen Eculizumab verwendet werden. Patient:innen mit therapierefraktären Formen einer MPGN sollten möglichst in Studien eingeschlossen werden.

## Rekurrenz nach Nierentransplantation

Das Management einer Nierentransplantation bei Patient:innen mit einer MPGN ist herausfordernd. Das Risiko einer Rekurrenz der Grunderkrankung ist ähnlich hoch bei einer IK-GN (42–53 %), einer C3G (60–86 %) und einer DDD (56–86 %), und ist mit einem reduzierten Transplantüberleben assoziiert (zusammengefasst in [1]). Aufgrund des hohen Relapsrisikos und des damit verbunden erhöhten Risikos das Transplantat zu verlieren, besteht kein Vorteil einer Lebendspende. Das hat viele Transplantationszentren dazu veranlasst, eine Nierenlebendspende bei einer MPGN, ähnlich wie auch bei einer primären FSGS, die auch mit einem hohen Relapsrisiko nach einer Transplantation assoziiert ist, kritisch zu sehen. Zu den Risikofaktoren für eine Rekurrenz der Grunderkrankung zählen erniedrigtes C3 bei der Transplantation, RPGN sowie Halbmonde in der Eigennierenbiopsie, Lebendspende von einem Verwandten, Vorhandensein eines monoklonalen Proteins, und eine präemptive Nierentransplantation [23].

Nachdem eine Rekurrenz der MPGN unter Immunsuppression auftritt, die üblicherweise Steroide und MMF beinhaltet, sind die therapeutischen Optionen eingeschränkt. Während milde Verläufe (stabile Nierenfunktion, nicht-nephrotische Proteinurie) optimal supportiv behandelt werden sollten, sollten moderate und schwere Verläufe entsprechend aggressiv therapiert werden. Zu den Optionen zählen Rituximab, Eculizumab, Cyclophosphamid und PLEX, wobei die Indikation, die Intensität als auch die Dauer der Therapie aufgrund der limitierten Evidenzlage individuell entschieden werden müssen.
